# Prediction Performance Comparison of Risk Management and Control Mode in Regional Sites Based on Decision Tree and Neural Network

**DOI:** 10.3389/fpubh.2022.892423

**Published:** 2022-05-26

**Authors:** Wenhui Zhu, Jun He, Hongzhen Zhang, Liang Cheng, Xintong Yang, Xiahui Wang, Guohua Ji

**Affiliations:** Center for Soil Protection and Landscape Design, The Innovation Center of Zero-Waste Society, Chinese Academy of Environmental Planning, Beijing, China

**Keywords:** decision tree (DT), artificial neural network (ANN), regional sites, risk management and control mode (RMCM), prediction performance

## Abstract

The traditional risk management and control mode (RMCM) in regional sites has the defects of low efficiency, high cost, and lack of systematism. Trying to resolve these defects and explore the application possibility of machine learning, a characteristic dataset for RMCM in regional sites was established. Three decision tree (DT) algorithms (CHAID, EXHAUSTIVE CHAID, and CART) and two artificial neural network (ANN) algorithms [back propagation (BP) and radial basis function (RBF)] were implemented to predict RMCM in regional sites. The results showed that in the aspects of accuracy (ACC), precision (PRE), recall ratio (REC), and *F*_1_ value, CART–DT was superior to CHAID–DT and EXHAUSTIVE CHAID–DT (E-CHAID–DT); and BP–ANN was superior to RBF–ANN. However, CART–DT was inferior to BP–ANN in ACC, PRE, REC, and *F*_1_ value. BP–ANN model is good at non-linear mapping, and it has a flexible network structure and a low risk of over-fitting. The case study of a typical county demonstration area confirmed the extensibility of the method, and the method has great potential in RMCM prediction in regional sites in the future.

## Introduction

Risk management and control technology for contaminated sites is a highly adaptive risk-based management method involving physical/chemical/biological remediation technology, engineering control, and institutional control measures (1, 2). Systematic integration of risk control technology and methods in the early stage of contaminated sites and determination of an effective risk control mode can further reduce costs and uncertainties in the implementation process (3). The common risk management and control mode (RMCM) mainly includes the following four types: (1) Remediation; (2) institutional control; (3) engineering control and institutional control; (4) remediation, engineering control, and institutional control. The determination of the best RMCM is essential for the environmental management in sites, and the extensive field surveys will consume a lot of time and resources, so the screening methods of regression model or empirical model have been widely studied.

The traditional risk management mode in sites mainly adopts a regression model or empirical model, including screening matrix (4, 5), analytic hierarchy process (AHP) (6, 7), elimination et choix traduisant la realite (ELECTRE) (8, 9), technique for order preference by similarity to ideal solution (TOPSIS) (10), preference ranking organization method enrichment evaluation (PROMETHEE) (11, 12), Gray evaluation (GE) (13), non-additive measure (14, 15), and the combination of these methods. Although these methods are different, they cannot solve complex non-linear relationships in essence, and the application of these methods is often based on a certain cognitive level of causality (11). Regional sites involve a large number of plots with complex types and different site conditions. Decision-makers often lack the understanding of causality, and the datasets that affect the determination of RMCM often have complex non-linear relationships. Once the screening method of regression model or empirical model is used, it is inefficient and costly, and it cannot effectively solve the problem. It needs to be further explained that, even if the parameters involved in regional sites can be measured, the traditional regression model or empirical model cannot use all available relevant factors to establish an appropriate prediction model. In fact, data mining methods can be used to solve this problem. The survey of some construction land in 73 sub-sectors of the National Economic Industry Classification (GB/T 4754-2017) has been completed, but the risk of land use in more than 1,000 sub-sectors is unknown. The problem of site pollution in China will show a rapid growth trend in the follow-up period, and the trend of site regional pollution will become more and more obvious. In the future, pollution investigation and risk control of new construction land and large number of stock construction land at the regional scale will still be a great challenge for local ecological environment authorities. The realization of intelligent management and control decision of regional-scale sites is the key development direction of site management in the future. The intelligent management and control decision of regional-scale sites can provide fast, accurate, and efficient top-level design of regional planning for the risk control of new construction land and a large stock of construction land in the future, so as to improve the efficiency and level of local government site management.

In recent years, big data technology has been widely used in the field of environment (16–18), especially the application of machine learning method provides a new idea for the intelligent decision-making of RMCM in regional sites. Decision tree (DT) and artificial neural network (ANN) are commonly used supervised machine learning algorithms, which can realize the mining and classification management of complex non-linear relationship datasets without causal cognition (19). Therefore, DT and ANN were used to conduct extensive research to predict air environmental quality (20–24), diagnostic optimization and prediction of chemical processes (25–29), soil and groundwater pollution prediction (30–32). The existing research on DT and ANN algorithms mainly focuses on two aspects. The first one is to analyze the training set and explore the internal structural relationship of data to construct the classification model. The other one is to use the test set to test the classification model and examine the generalization ability of the classification model. Although DT and ANN algorithms have been applied in the field of soil and groundwater pollution prediction, there are few reports on early decision-making of risk control mode in regional sites. In addition, the index system constructed by traditional methods is mainly aimed at specific sites which lack regional system considerations, and it is difficult to provide reference for early decision making of risk control mode in regional sites.

The decision-making of site pollution is very professional, which requires a large number of phased surveys and risk assessments as support. The state and the local governments cannot achieve accurate decision-making with sufficient funds, energy, and time in a short period of time according to the professional methods stipulated in the guidelines, so the rapid decision-making method that managers want is born. Aiming at the defects of low efficiency, high cost, and the lack of systematism in the determination of traditional RMCM in sites; this study constructed the decision index system and dataset of RMCM in regional sites and used different DT algorithms (CHAID, E-CHAID, and CART) and different ANN algorithms [(back propagation (BP) and radial basis function (RBF)] to explore the feasibility of the two types of machine learning algorithms applied to the prediction of RMCM in regional sites, providing a reference for early management decisions in regional sites.

## Models and Methods

### Research Approach and Technical Route

**Figure 1:**
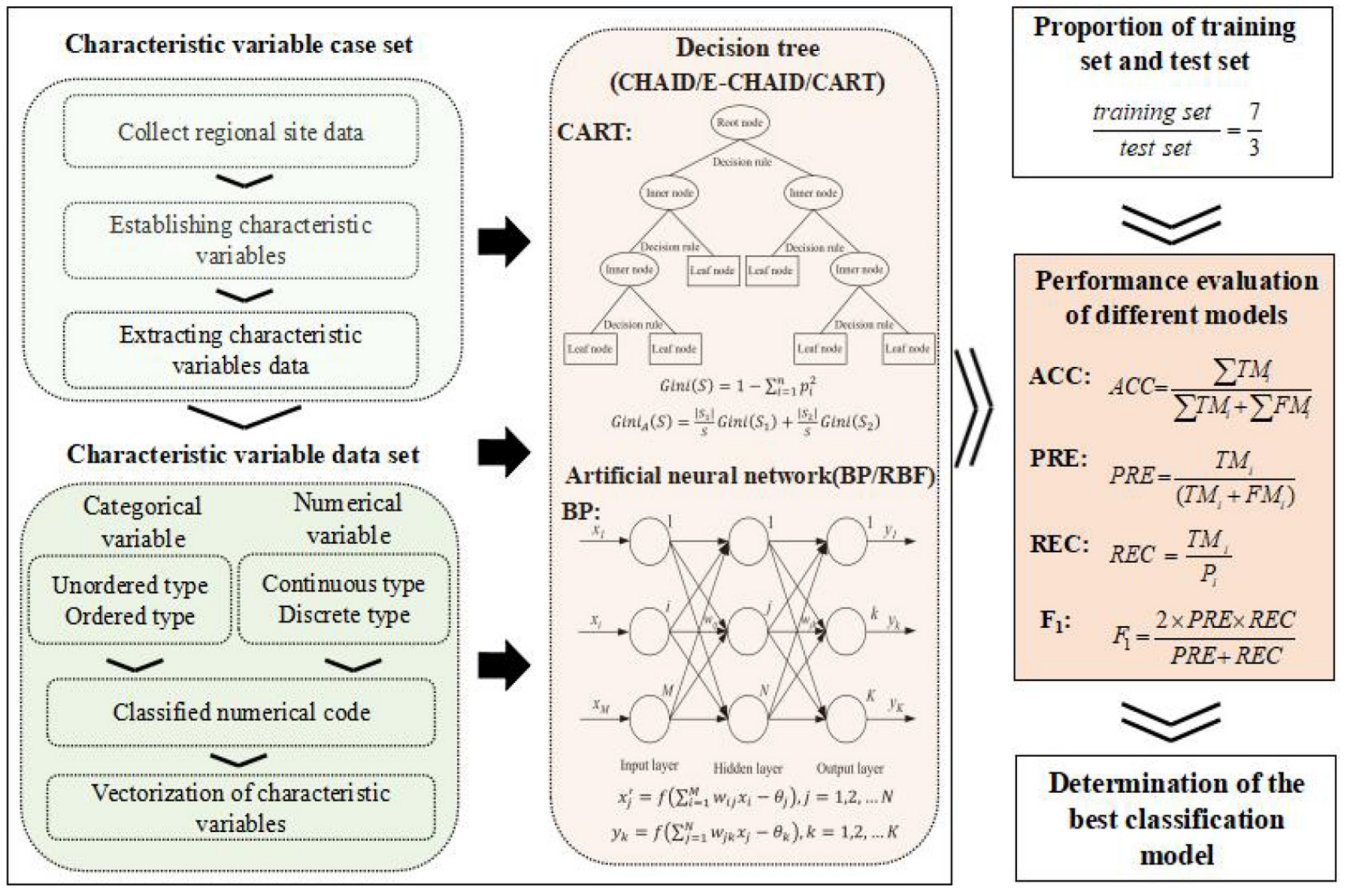
Technical route of RMCM prediction in regional sites.

The technical route of RMCM research in regional sites is shown in Figure 1.

It can be seen from Figure 1 that this study consisted of the following three main steps: (1) The case set of characteristic variables was constructed by data collection, characteristic variable construction, and data extraction. The data sources of this study include the recommended system risk management and remediation scheme of contaminated sites and the project information of additional regions or basins. The collected data were used to construct 20 characteristic variables (Table 1), and the relevant information of each case was further extracted by means of natural language processing. The case set of characteristic variables was constructed by expert decision judgment. (2) The characteristic variables were vectorized, and the characteristic variables dataset was constructed. The variable types of the characteristic variables in the case set are complex, including unordered categorical variables, ordered categorical variables, and continuous and discrete numerical variables. Numerical codes of different types of variables were vectorized to further improve the applicability of the model and reduce the computational complexity (the vectorization rules of characteristic variables are described in Table 1), and 226 characteristic variable datasets were formed. Unordered categorical variables can only be measured in classification, with no order, rank, or size, and ordered categorical variables can be measured in index, attribute classification, order, and rank. The attribute classes of the unordered categorical variables and the ordered categorical variables can be numerically encoded directly. The original data of continuous and discrete numerical variables were classified into ordered categorical variables by expert decision, and then the numerical coding was carried out. (3) Classification modeling, model performance evaluation, and analysis were done. The different DT algorithms (CHAID, E-CHAID, and CART) and different ANN algorithms (BP and RBF) were used to explore the feasibility of two kinds of machine learning algorithms applied to RMCM prediction in regional sites from ACC, PRE, REC, *F*_1_, and other aspects.

**Table 1:** Description of vectorization rules of characteristic variables.

**Type**	**Variable**	**Description of variable classification**	Numerical code^*^
1	Regional dominant land type (RDLT)	Dominant land type: sensitive; non-sensitive	{0, 1}
	Regional land value-added potential (RLVP)	Land increment is higher or lower than restoration cost: yes; no	{0, 1}
	Regional dominant industry risk (RDIS)	Whether it belongs to smelting, petrochemical, coking, electroplating, tanning, hazardous waste disposal industries: yes; no	{0, 1}
	Regional pollution level (RPL)	≥ intervention value; screening value < X < intervention value	{0, 1}
	Regional dominant functional area (RDFA)	Ecological; agricultural; urban	{1, 2, 3}
	Regional protection goal (RPG)	Surface water; groundwater; people; other	{1, 2, 3, 4}
	Regional pollution type (RPT)	Heavy metal; organic; compound	{1,2,3}
	Regional topography (RT)	Plain; hill; mountain	{1, 2, 3}
2	Regional pollution range (RPR)	Large (non-point); medium (points); small (point)	{1, 2, 3}
	Regional groundwater migration (RGM)	Strong (gravels); medium (sand); weak (silt)	{1, 2, 3}
	Regional soil barrier capacity (RSBC)	Strong (clay); medium (sand or silt); weak (gravels)	{1, 2, 3}
3	Regional average production period (RAPP)	Long (>20 years); medium (5–20 years); short (<5 years)	{1, 2, 3}
	Regional per capita gross domestic product (RPCGDP)	Advanced (> $20000); medium ($8000–$20000); backward (< $8000)	{1, 2, 3}
	Regional road network density (RRND)	Large (>5 km/km^2^); medium (1–5 km/km^2^); small (<1 km/km^2^)	{1, 2, 3}
	Regional average annual rainfall (RAAR)	Humid (>800 mm); medium (400–800 mm); arid (0–400 mm)	{1, 2, 3}
	Regional average annual wind speed (RAAWS)	Strong (>4 m/s); medium (2–4 m/s); weak (<2 m/s)	{1, 2, 3}
	Regional cultivated land density (RCLD)	Large (>30%); medium (10–30%); small (<10%)	{1, 2, 3}
	Regional river network density (RRD)	Large (>0.5 km/km^2^); medium (0.1–0.5 km/km^2^); small (<0.1 km/km^2^)	{1, 2, 3}
4	Regional enterprise density (RED)	Large (≥ 5/km^2^); medium (1–5/km^2^); small ( ≤ 1/km^2^)	{1, 2, 3}
	Regional population density (RPD)	Large (>100 p/km^2^); medium (25–100 p/km^2^); small (<25 p/km^2^)	{1, 2, 3}

* *Type 1 represents unordered categorical variable; Type 2 represents ordered categorical variable; Type 3 represents continuous variable; Type 4 represents discrete variable*.

The case set data of this study mainly came from the regional or basin site project information carried out in Guangdong, Guangxi, Guizhou, Hunan, Hubei, Jiangsu, Zhejiang, Henan, Anhui, Hebei, Inner Mongolia, Qinghai, Gansu, and other provinces from 2010 to 2020. On the basis of the collection of case sets, 20 relevant index systems were determined by constructing the regional scale site RMCM, and the relevant information of each case is further extracted. With the help of an expert decision-making judgment method, different classification variables were classified and transformed, and 226 datasets that can be used for DT and neural network model training and testing were finally determined. The data of this study were calculated by IBM SPSS Modeler 18.0.

### The DT and ANN Models

The DT model is widely used in the field of data mining, which represents a mapping relationship between object attributes and object values. Its basic purpose is to divide the total research sample into several relatively homogeneous sub-samples through some characteristics. The values of internal dependent variables in each sub-sample are highly consistent, and the corresponding impurities are as far as possible between different sub-samples (33, 34). All DT algorithms follow this principle, and only the definition of impurities is different, such as using *p*- value, variance, information entropy, and the Gini index as the measurement index. The CHAID, E-CHAID, and CART are three typical DT algorithms. The CHAID and E-CHAID algorithms use the chi-square test as the basic method of tree classification, which can support the generation of multi-tree. The CART algorithm uses the Gini index as a tree classification method, which only presents a binary tree structure. The CHAID and E-CHAID algorithms are only applicable to the categorical variables, while the CART algorithm can deal with the case that the target variable is a continuous variable, which can effectively solve the problem of missing data in the analysis (35, 36). The CHAID, E-CHAID, and CART algorithms are all applicable to the dataset of this study.

As the most widely used multi-layer feed-forward network in ANN, BP, and RBF usually have a three-layer structure, namely an input layer, a hidden layer, and an output layer. However, BP is a topological network structure with a multi-layer hidden layer (37, 38). The training process of BP–ANN is mainly the forward propagation of information and the backward propagation of error. If there is a large error between the predicted results and the expected output, the error value will be transmitted backward along the network, and then the network weights and thresholds are adjusted. The internal structure of BP–ANN is simpler than that of RBF–ANN. When large-scale data are collaboratively processed by BP–ANN, the influence of single error data on the overall prediction results is small, which is the global approximation of non-linear mapping. The hidden node of RBF–ANN uses the distance between the input mode and the center vector (such as the Euclidean distance) as the independent variable of the function and uses the RBF as the activation function. The farther the input of neurons is from the center of the RBF, the lower the activation of neurons is. The output of RBF is closely related to the “local” hidden nodes near the input mode of the data center, so RBF–ANN has the characteristic of “local mapping.”

### Model Evaluation Methods

Due to the use of non-linear algorithms in machine learning algorithms, such as DT and ANN, it is difficult to use conventional statistical test methods to evaluate the performance of models. The confusion matrix between the real classification of datasets and the prediction classification of models can effectively evaluate the actual performance of models. The indicators that reflect the results of this confusion matrix often include accuracy (ACC), precision (PRE), recall (REC), *F*_1_ (harmonic average of PRE and REC), etc. as shown in formulas (1)–(4).


(1) $$\begin{array}{r} ACC=\frac{TM_{1}+TM_{2}+TM_{3}+TM_{4}}{\begin{array}{c} TM_{1}+TM_{2}+TM_{3}+TM_{4}+FM_{1}+FM_{2}\\ +FM_{3}+FM_{4} \end{array}} \end{array}$$

where ACC represents the percentage of RMCMs correctly classified; *TM*_1_, *TM*_2_, *TM*_3_, and *TM*_4_ are the numbers of samples predicted correctly in different RMCMs, respectively; *FM*_1_, *FM*_2_, *FM*_3_, and *FM*_4_ are the numbers of samples predicted incorrectly in different RMCMs, respectively.


(2) $$\begin{array}{r} PRE=\frac{TM_{i}}{\left(TM_{i}+FM_{i}\right)} \end{array}$$

where PRE represents the percentage of samples in a certain type of RMCMs to the samples predicted to be in that RMCM; *TM*_*i*_ is the number of samples predicted correctly in all samples predicted to be in that RMCM; *FM*_*i*_ is the number of samples predicted incorrectly in all samples predicted to be in that RMCM.


(3) $$\begin{array}{r} REC=\frac{TM_{i}}{P_{i}} \end{array}$$

where REC indicates the percentage of samples predicted correctly to be in a certain type of RMCMs to all samples in that RMCM; *TM*_*i*_ is the number of samples predicted correctly to be in a certain type of RMCMs; *P*_*i*_ is the number of all samples in that RMCM.


(4) $$\begin{array}{r} F_{1}=\frac{2\times PRE\times REC}{PRE+REC} \end{array}$$

where *F*_1_ is a comprehensive evaluation index representing the harmonic average of PRE and REC; the definition of PRE and REC are shown in formulas (2) and (3), respectively.

## Model Construction and Analysis

### The DT Construction and Analysis

**Figure 2:**
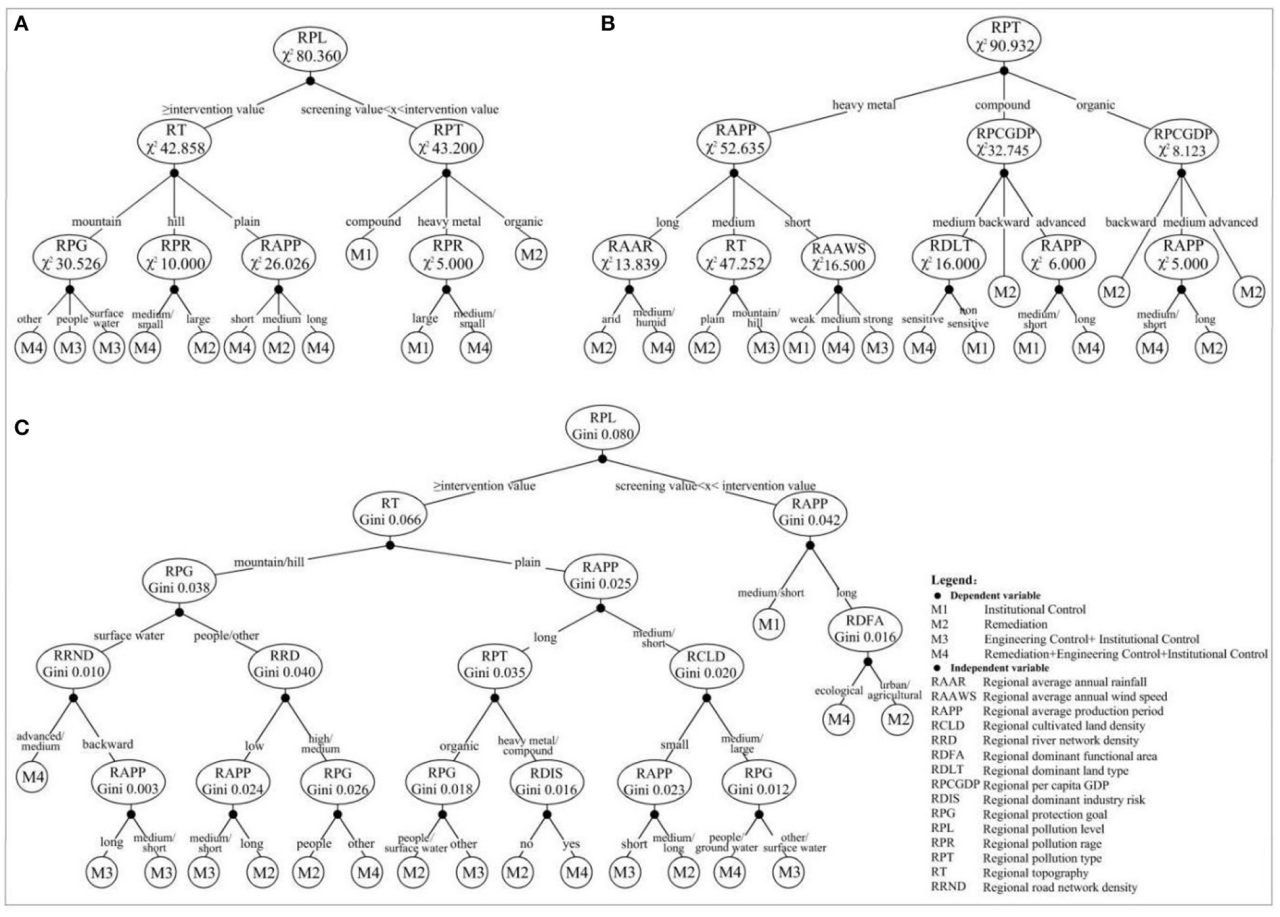
The DT model based on CHAID algorithm **(A)**, E-CHAID algorithm **(B)**, and CART algorithm **(C)**.

Different DT algorithms (CHAID, E-CHAID, and CART) introduced in Section The DT and ANN Models were applied to construct the DT models of RMCM in regional sites according to the technical route determined in Section Research Approach and Technical Route, as shown in Figure 2. The conditions for constructing the DT model were that the minimum number of cases of the parent node was 5 and the minimum number of cases of the child node was 1.

Figure 2 shows that CART-DT was significantly different from CHAID-DT and E-CHAID-DT in sufficient degrees in structure and growth. The CHAID and E-CHAID generate a multi-tree, while CART generates a binary tree. The fuller growth of CART-DT may be beneficial to the improvement of classification prediction performance. The maximum tree depth of CHAID-DT and E-CHAID-DT was 3, while that of CART-DT was 5; The numbers of growth nodes of CHAID-DT, E-CHAID-DT, and CART-DT were 19, 26, and 35, respectively, and the numbers of leaf nodes were 12, 16, and 18, respectively. In addition, the optimal segmentation variables and segmentation points of different DT algorithms were significantly different. The CHAID and E-CHAID algorithms use the chi-square test as the basic method of tree classification. The greater the chi-square value (χ2) is, the greater the deviation between the actual observed value and the theoretical inferred value is, and the higher the priority as the segmentation variable. The CART algorithm uses the Gini index (Gini) to measure the “impurity” of nodes, and the variable with the fastest decline of “impurity” is the variable of branch construction. The CHAID-DT root node divides the dataset into two relatively pure left and right sub-trees, “≥intervention value” and “screening value < X < intervention value,” respectively, with the largest characteristic variable “regional pollution level” of χ2 (80.36). The right tree was divided into four high-purity leaf nodes by RPT (χ^2^ = 43.20) and RPR (χ^2^ = 5.00). The left tree was divided into eight leaf nodes by RT (χ^2^ = 42.86), RPG (χ^2^ = 30.53), RPR (χ^2^ = 10.00), and RAPP (χ^2^ = 26.03). RPG, RPR, and RAPP are branches of RT. The E-CHAID-DT root node divides the dataset into three relatively pure left, neutron, and right sub-trees of “heavy metal,” “compound pollution,” and “organic matter” with the largest characteristic variable “regional pollutant type” of χ^2^ (90.93). The left tree was divided into seven leaf nodes by RAPP (χ^2^ = 52.65), RAAR (χ^2^ = 13.84), RT (χ^2^ = 47.25), and RAAWS (χ^2^ = 16.50). The neutron tree was further divided into five leaf nodes by RPCGDP (χ^2^ = 32.75), RDLT (χ^2^ = 16.00), and RAPP (χ^2^ = 6.00). The right tree was further divided into four leaf nodes by RPCGDP (χ^2^ = 8.12) and RAPP (χ^2^ = 5.00). The CART-DT root node divides the dataset into two relatively pure left and right sub-trees with “≥intervention value” and “screening value < X < intervention value” by the characteristic variable “regional pollution level” with the fastest decline of “impurity.” The left tree was further divided into 15 leaf nodes by the variables with the fastest decline in “impurity” (regional topography, RPG, regional enterprise production years, regional road network density, regional river network density, regional pollution type, RCLD, etc.). The right tree was further divided into three high-purity leaf nodes by the variables with the fastest decline in “impurity” (RAPP and RDFA). The lower the purity of leaf nodes is, the lower the prediction PRE, REC rate, and classification ACC of DT.

### The ANN Construction and Analysis

**Figure 3:**
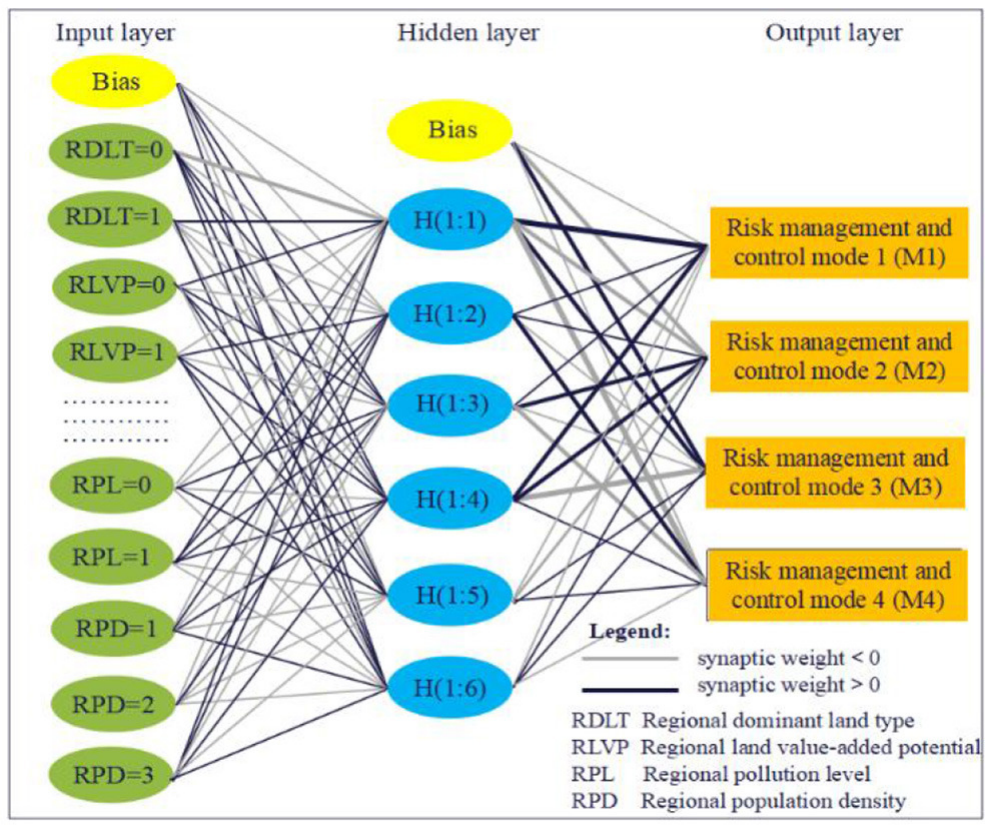
The ANN model based on the BP algorithm.

**Figure 4:**
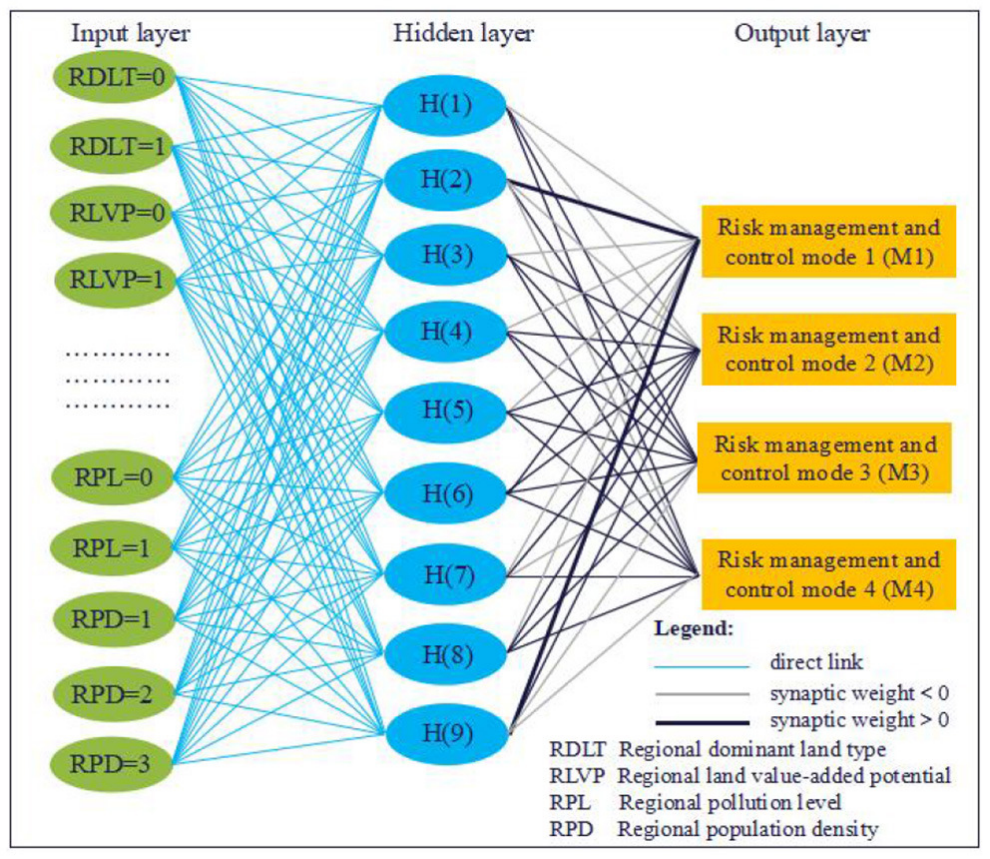
The ANN model based on the RBF algorithm.

Under the condition that the ratio of the training set to the test set was 7:3, different ANN algorithms (BP and RBF) and technical routes introduced in Materials and Methods were applied to construct ANN models for predicting RMCM in regional sites, respectively, as shown in Figures 3, 4.

## Materials and Methods

It can be seen from Figures 3, 4 that both RBF–ANN and BP–ANN are non-linear multilayer feedforward networks, but there are significant differences in network structure and training algorithm. In terms of the network structure, BP–ANN is the application of the gradient descent method in a multi-layer feedforward network, where synaptic weight connection is implemented between the input layer, hidden layer, and output layer units. While for RBF-ANN, a direct connection is implemented between the input layer and hidden layer units, and a synaptic weight connection is implemented between hidden layer and output layer units. In terms of the training algorithm, BP–ANN is to solve the minimum problem of the error function. The hidden layer adopts the hyperbolic tangent activation function, and the output layer adopts the softmax activation function. By using the forward propagation of information (randomly given weights and thresholds) and the backward propagation of error, the weights and thresholds of neurons in each layer are repeatedly corrected and adjusted in training until the error function drops to an acceptable level. The transformation of RBF–ANN from the input space to the hidden layer space is non-linear. The hidden node uses the distance function (Euclidean distance) as the basis function, and the hidden layer uses the standardized RBF (softmax) as the activation function so that the activation of all hidden units is standardized to 1. The input vector is directly mapped to the hidden space (without weight connection), and the output layer uses the softmax activation function.

## Results and Discussion

### Comparison of Prediction Performance of Different DT Algorithms

**Figure 5:**
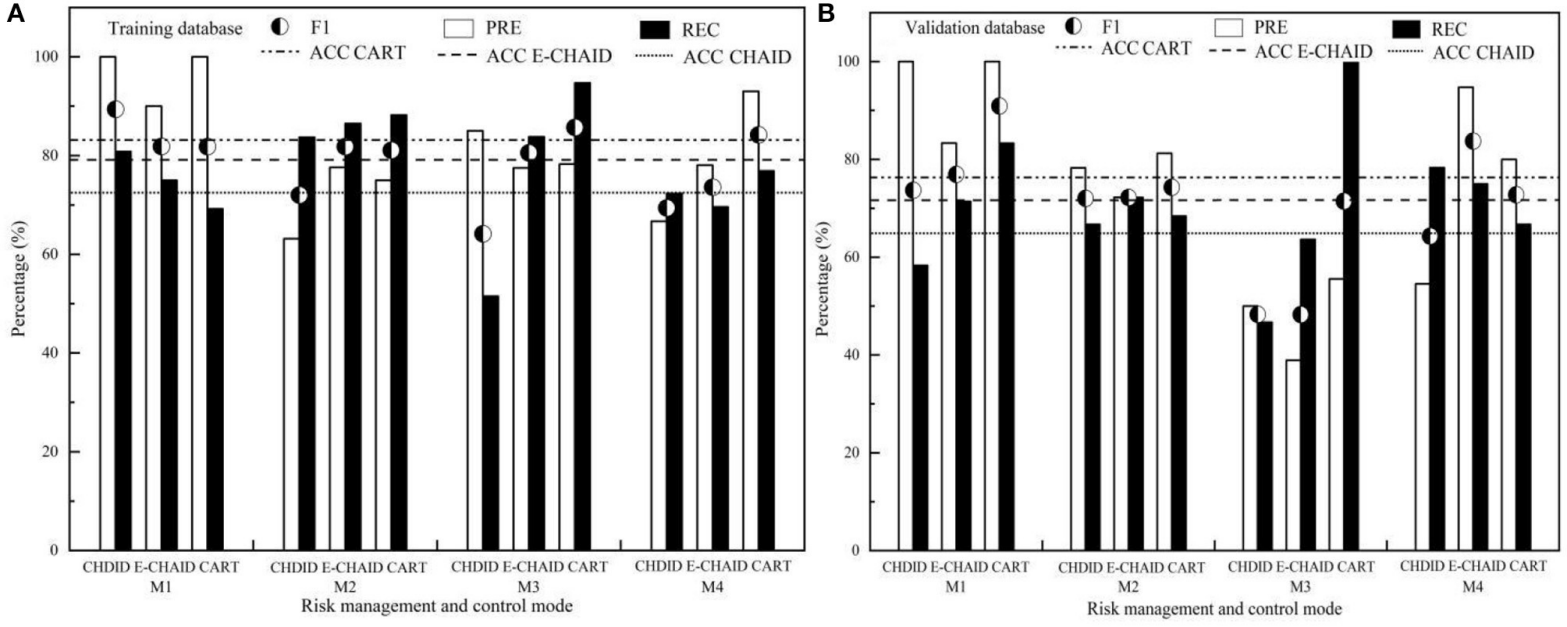
The prediction performance evaluation for DT of CHAID, E-CHAID, and CART [**(A)** training database; **(B)** validation database].

To evaluate the difference between the predicted classification and the real classification of the DT model constructed in Section The DT Construction and Analysis, ACC, PRE, REC, and *F*_1_ of different DT algorithms (CHAID, E-CHAID, and CART) are studied, as shown in Figure 5.

It can be seen from Figure 5 that CART-DT has higher ACC, equilibrated and stable PRE, REC, and F_1_ values, and it was better than CHAID-DT and E-CHAID-DT. The ACC of CART-DT training set and validation set (83.20, 76.30%) were better than those of E-CHAID-DT (79.20, 71.60%) and CHAID-DT (72.50, 64.90%). The PREs of CART-DT training set and validation set were generally superior to those of CHAID and E-CHAID algorithms, and CART-DT performance was relatively balanced. In the test set, the prediction PRE of the CART algorithm for M1 and M4 control modes (100.00, 93.02%) was better than that of the CHAID algorithm (100.00, 66.67%) and E-CHAID algorithm (90.00, 78.05%). The prediction PRE of the CART algorithm for M2 and M3 control modes (75.00 and 78.26%) was between that of the CHAID algorithm (63.16 and 85.00%) and that of the E-CHAID algorithm (77.59 and 77.50%). In the validation set, the prediction PRE of the CART algorithm for M1, M2, and M3 control modes (100.00, 81.25, and 55.56%) was better than that of the CHAID algorithm (100.00, 78.26, and 50.00%), and E-CHAID algorithm (83.33, 72.22, and 38.89%). The prediction PRE of the CART algorithm for M4 (80.00%) was between that of the CHAID algorithm (54.55%) and the E-CHAID algorithm (94.74%). The RECs of the CART-DT training set and validation set were equilibrated, better than those of CHAID and E-CHAID algorithms. In the training set, the proportions of correct prediction of M2, M3, and M4 by the CART algorithm were 88.20%, 94.70, and 76.90%, respectively, which were better than those of CHAID (83.70, 51.50, and 72.30%) and E-CHAID (86.50, 83.80, and 69.60%), but the proportion of correct prediction of M1 (69.20%) was slightly lower than that of CHAID and E-CHAID (80.80 and 75.00%). In the validation set, the proportions of correctly predicted M1 and M3 control modes by the CART algorithm were 83.30 and 100.00%, respectively, which were better than those of CHAID (58.30 and 46.70%) and E-CHAID (71.40 and 63.60%). The proportion of M2 correctly predicted by the CART algorithm was 68.40%, which was between CHAID (66.70%) and the E-CHAID algorithm (72.20%). The proportion of M4 correctly predicted by the CART algorithm was 66.70%, slightly lower than that of CHAID (78.30%) and E-CHAID (75.00%). The comprehensive index *F*_1_ reflects the relationship between PRE and REC rate, it showed that the prediction performance of the CART algorithm for different control modes (M1, M2, and M3) (81.80, 81.07, and 85.70% for training set, respectively, and 90.89, 74.27, and 71.43% for validation set, respectively) was better than that of CHAID and E-CHAID algorithms. The *F*_1_ value of training set M4 (84.20%) was also better than that of CHAID and E-CHAID algorithms, and only the *F*_1_ value of validation set M4 (72.75%) was between that of CHAID and E-CHAID algorithms.

### Comparison of Prediction Performance of Different Neural Network Algorithms

**Figure 6:**
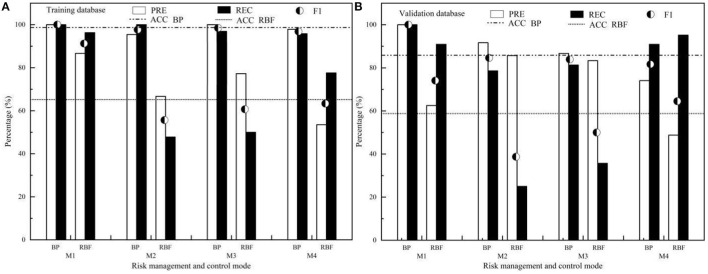
The prediction performance evaluation for ANN of BP and RBF **(A)** Training database; **(B)** Validation database.

To evaluate the difference between the predicted classification and the real classification of the ANN model constructed in Section The ANN Construction and Analysis, ACC, PRE, REC, and *F*_1_ of different ANN algorithms (BP and RBF) were studied, as shown in Figure 6.

Figure 6 shows that ACC, PRE, REC, and *F*_1_ values of the training set and validation set of BP–ANN were significantly better than those of RBF–ANN. The ACC of BP–ANN test set and the validation set were 98.00 and 85.70%, respectively, which were significantly better than 66.00 and 58.60% of RBF–ANN. The PREs of BP–ANN test set and validation set were 95.45–100.00% and 74.07–100.00%, respectively, which were significantly better than those of the RBF–ANN test set (53.52–86.67%) and validation set (48.78–85.71%). The RECs of the BP–ANN test set and validation set were 95.80–100.00% and 78.60–100.00%, respectively, which were significantly better than those of the RBF–ANN test set (47.80–96.30%) and validation set (25.00–95.20%). From the comprehensive index, *F*_1_, reflecting the relationship between PRE and REC, it can be seen that the *F*_1_ values of BP–ANN (96.82–100.00% for the test set, 81.63–100.00% for the validation set) were significantly better than those of RBF–ANN (55.68–91.23% for test set, 38.71–74.07% for the validation set), and BP–ANN model has better prediction performance and stability.

### Difference Analysis of Prediction Performance of Different Algorithms

As shown in Figure 5, CART-DT has higher ACC, equilibrated and stable PRE, REC, and *F*_1_ values, and it was better than CHAID-DT and E-CHAID-DT. The CHAID and E-CHAID algorithms adopt the local optimum principle, which means the nodes are not coherent. After a node is determined, the following growth process is completely carried out within the node. The CART focuses on the overall optimization, which means at first, the tree can grow as much as possible, and in the growth process of the tree, the same independent variable can be used repeatedly. The overall optimization algorithm of CART may be one of the best algorithms for DT to better solve the prediction performance of regional control mode.

As shown in Figure 6, ACC, PRE, REC, and *F*_1_ of the training set and test set of BP–ANN were significantly better than those of RBF–ANN. Both RBF–ANN and BP–ANN were non-linear multilayer feedforward networks (Figures 3, 4), but the algorithm was essentially different. One or more adjustable parameters (weights or thresholds) on BP–ANN affect any output and BP–ANN is the global approximation network, while RBF–ANN has only a few connection weights affecting the output for a local area of the input space and RBF–ANN belongs to the local approximation network. The essential difference between these two algorithms may be the main reason for the performance difference between RBF–ANN and BP–ANN in predicting the risk control mode in the regional site. In addition, although the numerical coding extends the value of discrete features to the Euclidean space, there may be a deviation in the calculation of RBF Euclidean distance. The direct connection between the input layer and the hidden layer of the RBF algorithm does not introduce weight, which is easy to distort and leads to lower prediction performance. The input layer—hidden layer–output layer—of the BP algorithm introduces weights, which can reduce the influence of numerical coding on classification performance, which may also be one of the reasons that affect the classification performance of the model.

This study found that the classification performance of BP–ANN for RMCM in regional sites was better than that of CART-DT, but there was no significant improvement. The ACC values of the BP–ANN test set and the test set were 98.00 and 85.70%, respectively; the ACC values of the CART-DT test set and the test set were 83.20 and 76.30%, respectively. Existed studies have shown that DT is suitable for small data, and BP–ANN is more suitable for large data. Generally, BP–ANN uses 500–1,000 data samples (19), but there is no clear quantitative limit. The CART-DT dataset achieved relatively good prediction performance, but the classification prediction was, obviously, laborious and needed pruning. With the increase in the number of datasets in the future, the generated DT model will become more and more complex, the over-fitting risk will increase, and the improvement of prediction performance will be limited. Although BP–ANN in this study achieved better classification results than CART-DT, the application of BP–ANN has not fully demonstrated its strong non-linear classification advantages because of the limitation of the dataset. With the growth of the future regional site dataset, BP–ANN has great potential for predicting the future regional site risk control model. The BP–ANN can not only be used to predict the RMCM in regional sites but also be applied to the classification of pollution identification and risk management and control effectiveness prediction, and the rapid screening of specific land control technology in regional sites.

The DT and ANN were both mature methods, and different data processing methods may affect the prediction results. In this study, based on the numerical coding method, the data preprocessing was realized by using the classification coding method. The influence of different types of data preprocessing methods on prediction results needs further study.

### Analysis of the Impact of Input Variables on Output Performance

**Figure 7:**
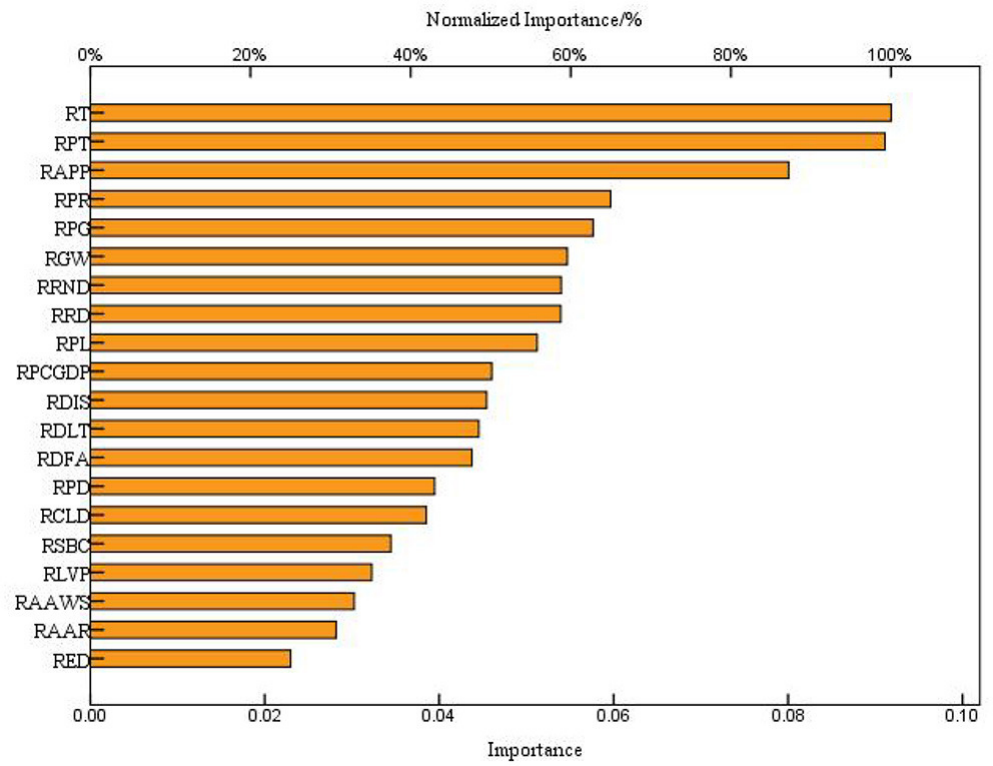
The importance of the contribution rate of BP–ANN input neurons to output variables.

Analyzing the difference in prediction performance of different algorithms showed that BP–ANN has the best performance in predicting RMCM in regional sites. Figure 7 shows the importance of influencing the contribution rate of BP–ANN input neurons to output variables.

It can be seen from Figure 7 that the relative importance of RT, RPT and RAPP to BP–ANN output was very high, reaching 100.00, 99.30, and 87.20%, respectively. The RPR, RPG, RGM, RRND, RRD, RPL, RPCGDP, RDIS, RDLT, RDFA, RPD, and RCLD were relatively important to the output of BP–ANN, which were as high as 65.00, 62.80, 59.60, 58.80, 58.70, 55.80, 50.10, 49.50, 48.50, 47.60, 43.00, and 42.00%, respectively. The RSBC, RLVP, RAAWS, RAAR, and RED also contributed to the relative importance of BP–ANN output, which were 37.50, 35.20, 33.00, 30.70, and 25.00%, respectively. The traditional RMCM adopts the method of decision screening by block, and the prediction of RMCM in regional sites based on BP–ANN regarded the regional sites as a whole, and the RMCM was comprehensively determined by different types of regional environmental factors. Figure 7 depicts the influence of input variables on the prediction results, which showed that the number of traditional sites was not the main factor affecting the BP–ANN prediction of RMCM, and RT, RPT, and RAPP were the main factors affecting decision-making.

### Case Study of Typical County-Level Demonstration Areas

#### Overview of the Study Area

**Figure 8:**
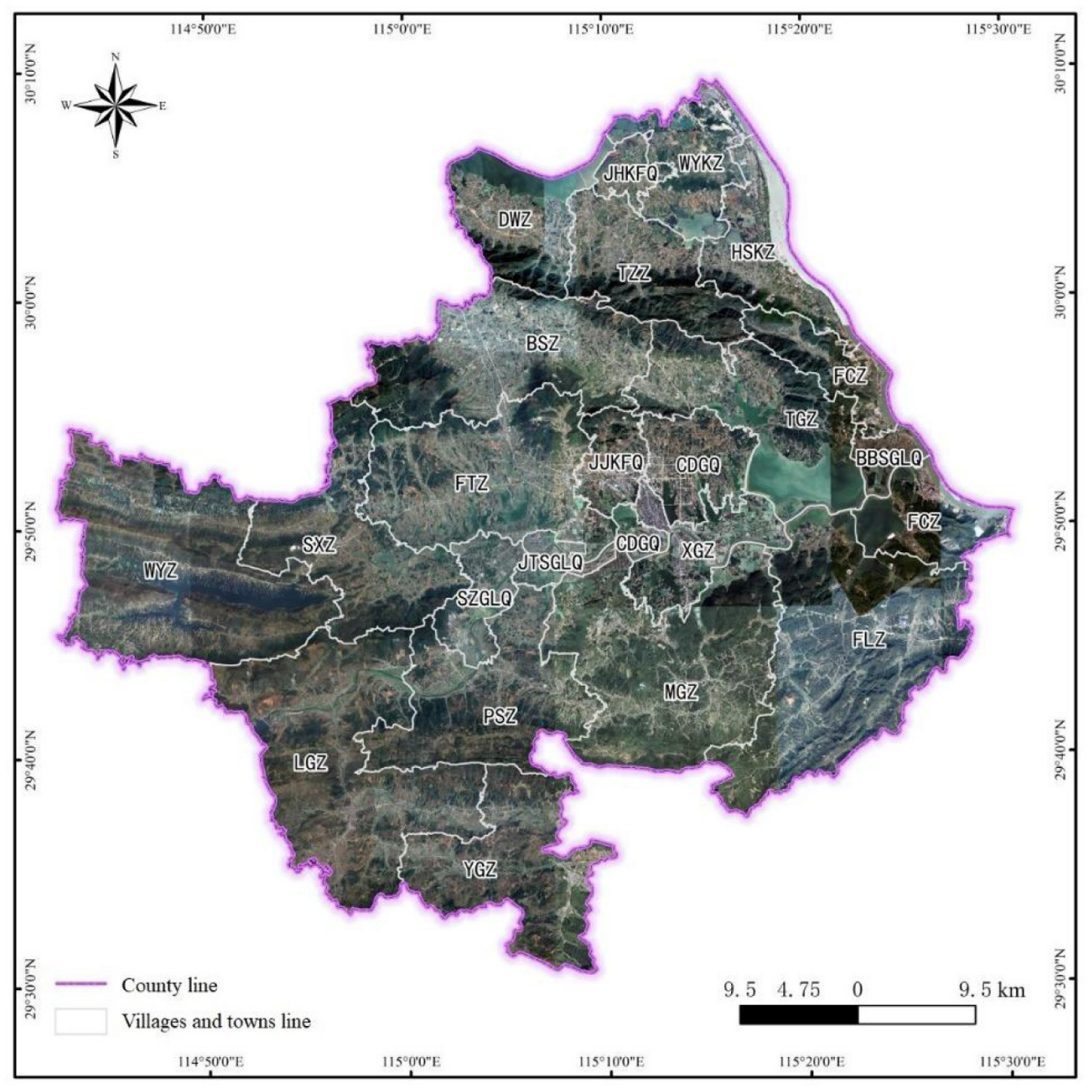
Schematic diagram of geographical location of the study area.

The study area was located in a city in central China, with geographical coordinates between 29°30′35″-30°9′14″N and 114°43′3″-115°30′12″E. The geographical location of the study area is shown in Figure 8.

The study area covers an area of 2,780 km^2^, of which the agricultural land accounts for 88.08%, the construction land accounts for 8.34%, and the non-use land accounts for 3.58%. The gross domestic product (GDP) of the whole county of China was 28.3 billion yuan, and the output value of industrial enterprises above the designated size was 14.457 billion yuan. Among the industries, the industrial output value of non-ferrous metal mining and dressing, chemical raw materials and chemical products manufacturing, and metal products industry was 2.337 billion yuan, accounting for 16.63% of the total industrial output above the designated size.

#### Data Source and Pre-Treatment

By means of data collection, interview, and field survey, the data of 20 characteristic variables in the study area were obtained. Table 2 shows the data sources.

**Table 2:** Statistical list of data sources.

**Serial number**	**Characteristic variable name**	**Data source**
1	RT	Digitization of Geomorphologic Map of China (scale 1:4 million)
2	RAAR	Spatial interpolation dataset of annual precipitation in China in 2015, with an ACC of 1 km ×1 km
3	RAAWS	Average the wind speed statistics of meteorological stations in the study area (2015)
4	RCLD	Data of the Second National Land Use Survey−2016 National Land Change Survey
5	RRD	National 1:1 million basic geographic database (2015)
6	RPT	Statistics of land survey results of enterprises in key industries in the study area
7	RPL	Census data of soil pollution in the study area
8	RPR	Census data of soil pollution in the study area
9	RSBC	Spatial distribution data of soil texture in China (1:1 million)
10	RGW	Hydrogeological map of China (1:6 million)
11	RPCGDP	China's km grid gross domestic product (GDP) distribution dataset (2015), with an ACC of 1 km ×1 km
12	RLVP	Comprehensive judgment on the results of the urban master plan (2014–2030) in the study area and the results of personnel interviews.
13	RDFA	Achievements of the implementation plan of “three lines and one list” ecological environment zoning management and control in the study area
14	RDLT	Data of the Second National Land Use Survey-2016 Land Change Survey
15	RED	Statistics of land survey results of enterprises in key industries in the study area
16	RPD	China's population spatial distribution kilometer grid dataset, 2015, with an ACC of 1 km ×1 km
17	RRND	National 1:1 million basic geographic database (2015)
18	RDIS	Statistics of land survey results of enterprises in key industries in the study area
19	RPG	Land use survey of enterprises in key industries in the study area-statistical analysis of basic information collection results, supplemented by personnel interviews and on-site reconnaissance and judgment.
20	RAPP	Land use survey of enterprises in key industries in the study area—statistics of basic information collection results

After obtaining the basic data of 20 characteristic variables in the study area, two steps of data preprocessing were taken: (1) The statistics of partition attributes of different categories of vectorized data. For continuous and discrete vector data, the functions of the spatial analysis module in ArcGIS, such as partition statistics, area tabulation, spatial correlation, intersection analysis of the analysis module, geometric calculation of vector data attribute table, etc. were used to calculate the arithmetic average or additive average of each partition and realize partition attribute counting; after further qualitative judgment by expert decision, the vector data of unordered variables and ordered classified variables can realize partition attribute discrimination. (2) The numerical coding of different types of variables. The numerical coding was carried out for the classification of characteristic variables formed by partition attribute statistics to form datasets; attribute categories of unordered classified variables and ordered classified variables were directly coded; partition data of continuous and discrete numerical variables were classified into classified variables by expert decision, and then coded.

#### Prediction of Control Mode

**Figure 9:**
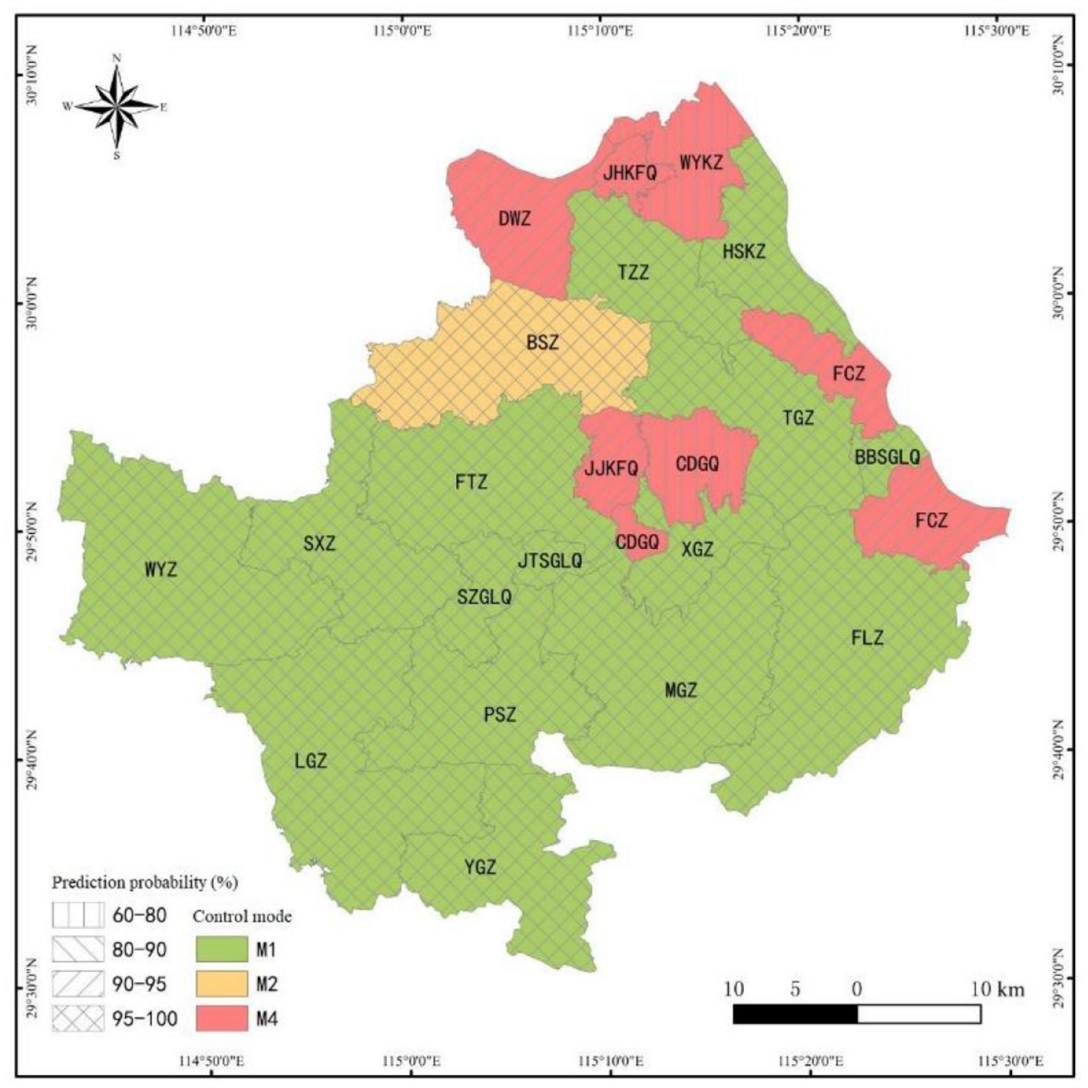
Schematic prediction results of RMCM in the study area (M1: institutional control; M2: remediation; M4: remediation, engineering control, and institutional control).

Based on the data of 20 characteristic variables collected in the study area, using the best DT algorithm decision rules selected, the RMCM of 22 towns/districts in the study area was predicted, and the prediction results (see Figure 9).

As can be seen in Figure 9, from the prediction results of the overall RMCM, the prediction ratio of different RMCMs in the study area was M1 > M4 > M2. The prediction of RMCMs in 22 towns/districts in the study area was mainly “institutional control” (15, accounting for 68.18%), followed by “remediation, engineering control, and institutional control” (6, accounting for 27.27%), and finally “remediation” (1, accounting for 4.55%). Judging from the prediction probability of different RMCMs, the prediction probability of “institutional control” and “remediation” was higher, followed by “remediation, engineering control, and institutional control.” The decision rules of “institutional control” and “remediation” were not easily influenced by the decision rules of other RMCMs, and the prediction probability was high, reaching 100%. For example, XGZ, TZZ, SXZ, LGZ, PSZ, MGZ, FLZ, WYZ, SZGLQ, BBSGLQ, and JTSGLQ were generally light in pollution and short in production history, and the prediction probability of recommending “institutional control” RMCM was 100%. The average prediction probability of the management and control mode of “remediation, engineering control, and institutional control” was 83.50%, which was lower than that of “institutional control.” The main reason for the decline was the influence of many decision rules of “engineering control and institutional control.” For example, CDGQ gathers chemical and smelting industries, with serious pollution exceeding the standard and many sensitive points in the region. In addition, the soil clay-particle ratio has a certain barrier performance, and the groundwater was poor in water abundance and mobility. The prediction probability of “remediation, engineering control, and institutional control” was only 60%, and the remaining 40% probability was controlled by the decision rules of “engineering control and institutional control.”

From the perspective of the concentrated distribution of non-ferrous minerals in the study area, the prediction probability of RMCM in the concentrated distribution area of non-ferrous minerals was high. Non-ferrous metal minerals in the study area were concentrated in BSZ-FTZ-JJKFQ-TGZ-FCZ. Also, JJKFQ and FCZ were affected by the long-term mining and smelting of non-ferrous metals, the pollution exceeded the standard seriously, and there were many sensitive points around them. Therefore, the prediction probability of “remediation, engineering control, and institutional control” was 94%. Also, TGZ and FTZ have light pollution degrees, and their production history was at a medium/short level. The prediction probability of recommending “institutional control” mode was 100%. Although the pollution degree of BSZ was light, it has a long production history, and the cultivated land density and human settlement density around the construction land were high. Simple institutional control may bring great risks to sensitive targets. In addition, the characteristics of this area with a small pollution range and medium GDP per capita suggest that the prediction probability of adopting the RMCM of “remediation” in local polluted areas was 100%. Judging from the distribution of energy, mineral, and coal in the study area, the prediction probability of RMCM in the energy, mineral, and coal distribution area was higher. Mineral coal was mainly distributed in the south (YGZ) and north (JHKFQ) of the county. Also, YGZ has a light pollution degree and a short production history, so the prediction probability of recommending the “institutional control” mode was 100%; JHKFQ mining has a long history of serious pollution and highly cultivated land density. The prediction probability of “remediation, engineering control, and institutional control” was 94%. From the perspective of limestone mineral distribution, the prediction probability of RMCM in a simple limestone mining area was high, but some areas were affected by the chemical industry, and the prediction probability of RMCM decreased. Limestone for cement and limestone for flux were mainly distributed in the eastern WYKZ-HSKZ-FCZ area. Moreover, HSKZ was engaged in limestone mining for a short time, and the pollution degree was light. The prediction probability of the “institutional control” mode was recommended to reach 100%. The mining history of WYKZ limestone was longer than that of Huangjiakou, and there were many chemical industry enterprises in the area, which leads to the compound pollution of heavy metals and organic compounds in the area, and the pollution exceeds the standard seriously. In addition, the potential of regional land appreciation was significantly higher than that of most other towns, so the probability of recommending the RMCM of “remediation, engineering control, and institutional control” was only 65% (the probability of recommending the RMCM of “engineering control and institutional control” was 35%).

## Conclusion and Suggestions

1. The performance of CART-DT in ACC, PRE, REC, and *F*_1_ was significantly better than that of CHAID-DT and E-CHAID-DT. The performance of BP–ANN in ACC, PRE, REC, and *F*_1_ was significantly better than that of RBF–ANN, but the prediction performance of CART-DT was significantly worse than that of BP–ANN. Also, BP–ANN has the best prediction performance in RMCM for regional sites.
2. The relative importance of 3 input variables (regional topography, regional pollutant type, and RAPP) to BP–ANN output was very high; 12 input variables (regional pollution range, RPG, RGM, etc.) were relatively important for BP–ANN output; Five input variables (regional soil barrier capacity, RVLP and regional average annual wind speed, etc.) also contribute to the relative importance of BP–ANN output.
3. The DT is very suitable for the condition of small data volume and multi-feature. With the increase of future data volume, the generated DT will become more and more complex, the over-fitting risk will increase, and the improvement of prediction performance will be limited. The BP–ANN model is good at non-linear mapping and has a flexible network structure and low risk of over-fitting, which has great potential in the RMCM prediction for regional sites in the future.
4. The environmental, economic, management, and other factors in different regions have a significant drive and hinder role for the quick decision site risk control mode for the local administrative department. Huge amounts of multi-source heterogeneous data acquisition are very important to intelligent control decision-making for regional sites, index system of intelligent control decisions for regional sites will also get improved with data accumulation. In the future, the breaking of data barriers between different management departments and the accumulation of more and more structured and unstructured regional data will provide a broader stage for the intelligent management and control decisions for regional sites.

## Data Availability Statement

The raw data supporting the conclusions of this article will be made available by the authors, without undue reservation.

## Author Contributions

WZ, XW, and JH contributed to conception and design of this study. WZ carried out the experiments, performed the statistical analysis, and completed the manuscript. HZ and LC made important contributions to the analysis and preparation of manuscripts. XY and GJ helped complete the analysis through constructive discussion. All authors contributed to manuscript revision, read, and approved the submitted version.

## Funding

The authors were financially supported by the National Key Research and Development Program of China (2018YFC1800200 and 2018YFC1800205).

## Conflict of Interest

The authors declare that the research was conducted in the absence of any commercial or financial relationships that could be construed as a potential conflict of interest.

## Publisher's Note

All claims expressed in this article are solely those of the authors and do not necessarily represent those of their affiliated organizations, or those of the publisher, the editors and the reviewers. Any product that may be evaluated in this article, or claim that may be made by its manufacturer, is not guaranteed or endorsed by the publisher.
